# Light-Time-Biomass Response Model for Predicting the Growth of Choy Sum (*Brassica rapa* var. *parachinensis*) in Soil-Based LED-Constructed Indoor Plant Factory for Efficient Seedling Production

**DOI:** 10.3389/fpls.2021.623682

**Published:** 2021-06-07

**Authors:** Jim Junhui Huang, Craig D’Souza, Weibiao Zhou

**Affiliations:** ^1^Environmental Research Institute, National University of Singapore, Singapore, Singapore; ^2^Department of Food Science and Technology, National University of Singapore, Singapore, Singapore

**Keywords:** choy sum, white LED light intensity, morphology, biomass, light-time-biomass response model, Akaike information criterion

## Abstract

Little is known about how exactly light plays its role in the growth of choy sum (*Brassica rapa* var. *parachinensis*), a widely cultivated vegetable in Asia. By applying a commercial soil using black peat as major constituent with 17:10:14 ratio of NPK fertilizer in this study, the growth responses of choy sum seedling to progressively increasing white LED light intensity in an indoor plant factory were investigated, where positive enhancements were observed in choy sum morphology and growth including both dry and fresh mass accumulation under higher light intensity till 400 μmol/(m^2^⋅s), then a reduction occurred due to light oversaturation and overheat. In indoor plant factory, the inhomogeneous distribution phenomenon of illumination level was inevitably occurred in indoor farm racks generally. For accurately evaluating the productivity of choy sum grown on such racks, a light-time-biomass response model of choy sum seedling grown at the seedling stage was thus established for the first time, which could reliably predict the production outcome of this species in indoor farming practice under various lighting condition and duration. The robustness of the model was further tested by model variation test and sufficient robustness of this model was confirmed. The new insight obtained for the light-dependence of choy sum growth and the light-time-biomass response model can be used to efficiently direct its seedling production in indoor plant factories.

## Highlights

- Light intensity significantly affects the morphology and biomass of choy sum seedling.
- Optimal range of white LED light intensity to grow choy sum seedling is around 400 μmol/(m^2^⋅s).
- Light-time-biomass response model developed can reliably predict choy sum dry biomass.

## Introduction

The consumption of vegetables has been an integral part of the human diet due to their many health-promoting functions, such as providing dietary fiber as roughage, a variety of bioactive substances, e.g., carotenoids, phenolic compounds, vitamins, and glucosinolates, contained under different levels, as well as their abilities to enhance satiety and promote the absorption of other macronutrients in the intestinal tract (Zhang et al., 2011; Samoulienë et al., 2013; Kwack et al., 2015; Walsh et al., 2015; Peluso et al., 2016; Padayachee et al., 2017; Yahia et al., 2017; Liang et al., 2018; Tan et al., 2020; Huang et al., 2021). However, traditional cultivation methods, which account for the bulk of vegetable production, tend to be inefficient and unreliable due to the constant fluctuations in sunlight, which is the main source of light energy for such methods (Alvino and Barbieri, 2016). For this reason, indoor plant factories were being developed for stable and large-scale vegetable productions, and playing an efficient solution for space research and closed agroecosystems in recent years (Fujiwara, 2019; Zhang et al., 2020).

Indoor plant factories are an attractive alternative by providing a better protection from pests and harsh external environmental conditions, or natural disasters. They also provide a means of controlling temperature and lighting conditions through the use of energy-efficient Light Emitting Diode (LED) or fluorescent lamps (Van Ieperen and Trouwborst, 2007; Fujiwara, 2019). However, the shelf surfaces of cultivation racks of indoor plant factories may undergo inhomogeneous distribution of illumination level due to the arrangement of lamps applied, leading to varied photosynthetic rates and consequently unpredictable productivities of vegetables grown on them. This could be owing to the lack of in-depth scientific study that reveals the relationship among plant productivity and illumination level, as well as cultivation duration. One way of solving this problem is by developing a reliable mathematical model correlating dry mass to light intensity and time corresponding to specific vegetables, to predict their respective productivities in indoor plant factory, which helps farmers to develop a strategy to maximize the efficiencies of photosynthesis and energy conversion in indoor plant factories.

The *Brassicaceous* vegetables such as choy sum, kai lan (*Brassica olearacea* var. *alboglabra*) and broccoli (*Brassica olearacea* var. *italica*) are popular and well-recognized for being rich in antioxidant substances such as vitamin C and E, carotenoids, and flavonoids, along with unique anti-carcinogenic bioactive substances like glucosinolates (He et al., 2000; Podsêdek, 2007; Liang et al., 2018). In particular, choy sum is widely cultivated and consumed in many Asian countries such as China, Japan, and Singapore, yet little information can be found with regards to its growth in response to light. In addition, the seedling stage of plant is of particular interest, it has been reported that lighting treatment during this growth period may significantly affect the subsequent development, growth and flowering of some plants at the transplantation stage (Pramuk and Runkle, 2005), indicating that the seedling stage of choy sum should be carefully studied as its light experience at this stage may significantly impact its later growth and development after being transplanted.

This study aimed to investigate the relationship between light intensity and duration versus various choy sum growth parameters, at the seedling stage in an indoor farm setting. It is hypothesized that there is an exponential/logistic relationship between choy sum biomass (dry weight in particular) and light intensity applied and elapsed time. In addition, a higher light intensity may positively influence choy sum seedling growth as long as it is below the light saturation point of this species.

## Materials and Methods

### Vegetable Species and Cultivation Conditions

Choy sum (*Brassica rapa* var. *parachinensis*) seeds were obtained from Ban Lee Huat Seed Pte Ltd. (Singapore). The standardized commercial substrate used for cultivating choy sum was purchased from Jiffy^®^ potting mix (Jiffy Florafleur 002 Universal potting soil; Toul, France), of which the major constituents are black peat with added fertilizer N+P+K as 17+10+14 and trace elements as stated by the producer. Cultivation was performed using 50-cavity germination trays, manufactured by Arianetech Pte Ltd. (Singapore). One seed was sowed in each cavity (0.05 m × 0.05 m) and placed under a custom-made indoor farm rack containing white LED tubes installed above each shelf on the rack. Each 22W LED tube applied consisted of 96 white LED chips.

**FIGURE 1:**
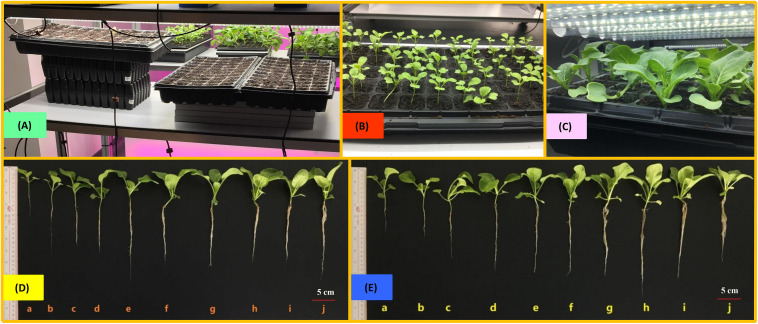
The pictures to show **(A)** the setting in this study, choy sum seedling grown **(B)** under low light intensity [50–150 μmol/(m^2^⋅s)] on day 14 and **(C)** under high light intensity [400–500 μmol/(m^2^⋅s)] on day 14 in the germination trays, as well as the morphologies of the representative choy sum seedlings grown under different white light emitting diode (LED) light intensities on **(D)** Day 14 and **(E)** Day 16, respectively (the transplantation days). Choy sum seedling grown under (a) 50 μmol/(m^2^⋅s); (b) 100 μmol/(m^2^⋅s); (c) 150 μmol/(m^2^⋅s); (d) 200 μmol/(m^2^⋅s); (e) 250 μmol/(m^2^⋅s); (f) 300 μmol/(m^2^⋅s); (g) 350 μmol/(m^2^⋅s); (h) 400 μmol/(m^2^⋅s); (i) 450 μmol/(m^2^⋅s); and (j) 500 μmol/(m^2^⋅s), respectively. The criterion for the representative seedlings to be presented in the singular photos of panels **(D,E)** were based on their average morphological data (Figure 4) under their respective light conditions.

Seedlings were cultivated under 10 levels of light intensities ranging from 50 ± 10 to 500 ± 10 μmol/(m^2^⋅s), with an interval of 50 μmol/(m^2^⋅s) between any two levels, by measuring the light intensity at the seedling canopy as the criterion. Different light intensities were achieved by varying the distance between the canopies of seedlings and the LED tubes, through cushioning the germination trays to different heights rooting from the layer surface of indoor farm rack, as shown in Figures 1A–C. In order to keep the fixed light intensity treating on the seedling canopy, the distance between the canopy of choy sum seedling and the LED tubes was fixed by gradually lowering the height of germination tray during the cultivation, according to the gradual increase of seedling canopy height. In this study, 200 μmol/(m^2^⋅s), the light intensity commonly applied in leafy vegetables indoors, was set as the control intensity (Colonna et al., 2016). The photoperiod was set at 12 h per day. Light intensity and spectrum were recorded using a light meter (ASENSE*TEK*^®^ Lighting Passport; Taiwan).

The cultivation temperature (°C) and environmental relative humidity (%RH) under all the tested light intensities, subjected to the distance between LED tubes and trays, were determined on day 16 also by using the above-mentioned light meter (with the functions of temperature and %RH measurements) and the installed site for determination was near the leaves of the seedlings, while temperature under dark condition was measured as 22.0 ± 2.0°C. The absolute humidity was calculated based on %RH and temperature as follows (Hall et al., 2016):

(1) $$\text{Absolute}\,\text{humidity}\,\left(\text{g}/{\text{m}}^{3}\right)=\frac{6.112\times e^{\left[\frac{17.67\,T}{T+243.5}\right]}\times \left(\%RH\right)\times 2.1674}{273.15+T}$$

Where *T* stands for temperature (°C).

The illumination conditions and white LED spectrum properties applied in this study were shown in Table 1. Irrigation was performed by adding 3.0 L of water to the trays through sub-irrigation method on the day of sowing (Day 0). Three respective irrigations of 1.0 L water were subsequently applied on Day 4, Day 10, and Day 14. Soil pH values under 50, 100, 200, 300, 400, and 500 μmol/(m^2^⋅s) were monitored daily by employing a soil direct pH tester (HI 981030, HANNA^®^ Instruments; Limena, Italy).

**TABLE 1:** Illumination conditions and white light emitting diode (LED) spectrum properties applied in this study.

Light intensity	Ultraviolet	Blue	Green	Red	Far red	Light energy provided	^*b*^*I*/*E*_*I*_ ratio
[μmol/(m^2^⋅s)]	(380–399 nm)	(400–499 nm)	(500–599 nm)	(600–700 nm)	(701–780 nm)	^*a*^*E*_*I*_ [J/(m^2^⋅s)]	(μmol/J)
50	0.04	15.22	22.65	12.94	1.08	11.13	4.49
100	0.06	28.94	46.02	26.00	2.11	22.88	4.37
150	0.12	43.48	68.51	38.77	3.31	34.01	4.41
200	0.16	58.04	91.18	51.18	4.40	44.91	4.45
250	0.19	73.19	113.21	63.72	5.27	56.29	4.44
300	0.24	87.16	136.47	76.85	6.26	67.81	4.42
350	0.23	101.00	159.52	89.82	7.30	78.68	4.45
400	0.28	117.86	181.02	101.54	8.11	90.14	4.44
450	0.40	134.02	202.89	113.80	9.40	101.14	4.45
500	0.37	148.36	225.24	126.93	10.42	111.79	4.47

*^*a*^*E*_*I*_ Is the light energy provided by the white LED light tubes.*

*^*b*^*I* Represents the light intensity.*

### Determination of Morphological Parameters and Biomass

The sampling days were based on various developmental stages of choy sum seedling, specifically the 1-, 2-, and 3-leaf stages. Additional sampling was carried out on Day 14 and Day 16 (set as transplantation day), respectively. On sampling day, choy sum seedlings together with the soil were carefully dug out from the tray cavity and transferred into a beaker of deionized water, to remove the soil with caution to avoid breaking off the roots. After the removal of soil and other attached impurities from the roots, choy sum seedlings were carefully dried using absorbent paper towels before the determination of morphological parameters and biomass. To determine the morphological parameters including shoot canopy height, hypocotyl diameter and length, leaf area, and root length, each seedling was laid on a black, matt surface and its photo was taken using the digital camera of iPhone 6S (iPhone, Apple Inc., Cupertino, CA, United States). Quantitative measurements of the morphological parameters were subsequently performed through image analysis using ImageJ 1.51j8 software (National Institute of Health; Bethesda, MD, United States).

For biomass assessment, the fresh weight of the whole seedling was firstly measured using an electronic weighing balance (Mettler Toledo ML303 Precision Balance; Greifensee, Switzerland). Afterward, its shoot and root were determined, respectively, by using a pair of scissors to separate the hypocotyl end and root. Subsequently, the shoot and root were lyophilized, and their dry weights were measured. The dry weight of the whole seedling was calculated by adding the dry weights of the shoot and root. The moisture content of the seedlings was determined as follows:

(2) $$\text{MCP}\left[\text{Moisture}\text{content}\text{percentage}\left(\%\right)\right]=\frac{F\,W-D\,W}{F\,W}\times 100\%,$$

Where FW stands for the fresh weight (mg/seedling) of choy sum seedling while DW represents the dry weight (mg/seedling) of the same choy sum seedling after lyophilization.

### Determination of Productivity

The biomass productivity in this experiment was calculated as follows:

(3) $$\text{PDY}\,\left(\text{Productivity}\right)\,\left[\text{g}\,/\,\left({\text{m}}^{2}\cdot \text{d}\right)\right]=\frac{M_{N}-M_{0}}{N},$$

Where *M*_*N*_ and *M*_0_ stand for the dry mass of seedlings per area (g/m^2^) measured on day *N* and day 0, respectively, and “*N*” represents the cultivation duration (d) (Huang et al., 2016). In this study, the productivity was calculated on the basis of germination tray (55 cm × 28 cm) we applied, with fixed planting density (50 cavities per tray). The footprint per choy sum seedling in the tray was 0.0025 m^2^ (0.05 m × 0.05 m) and 1 m^2^ of cultivation area (including tray margins) in the tray could grow up to 325 seedlings.

### Determination of Relative Growth Rate (μ)

The relative growth rate (RGR) on dry weight basis was calculated by the equation below:

(4) $$\mu \,\left[\left(\text{g}\,/\,\text{g}\right)\,/\,\text{day}\right]=\frac{L\,n\,D\,W_{N}-L\,n\,D\,W_{0}}{N},$$

Where *DW*_*N*_ and *DW*_0_ are the dry weight (mg) of the whole choy sum seedling measured on day *N* and day 0 under a given light condition, respectively. *N* represents the cultivation duration (d) (Tan et al., 2020).

### Determination of Photosynthetic Efficiency, Net Assimilation Rate and Net Photosynthetic Rate (*P*_*n*_, Light Response Model)

The photosynthetic efficiency is defined as follows:

(5) $$PE\left(\%\right)=\frac{E_{B}}{E_{\text{I}}}\times 100\%,$$

Where *E*_*B*_ stands for the free energy included in the dry biomass of choy sum shoot (edible portion), and *E*_*I*_ is the light energy in the spectrum of 380–780 nm including photosynthetically active radiation (PAR) range of 400–700 nm that was provided by the white LED light tubes as shown in Table 1. The light energy *E*_*I*_ [J/(m^2^⋅s)] based on light intensity [μmol/(m^2^⋅s)] was calculated as follows:

(6) $$E_{I}\,\left[\text{J}\,/\,\left({\text{m}}^{2}\cdot \text{s}\right)\right]=h\times c\times A\times 10^{3}\times \sum_{\lambda =380}^{\lambda =780}\frac{I_{\lambda }}{\lambda },$$

Where *h* stands for Planck constant (6.626 ×10^−34^J⋅s), *c* is light ray velocity (2.998 ×10^8^m/s), λ is the photon wavelength (nm), *I*_λ_ represents light intensity [μmol/(m^2^⋅s)] under certain wavelength from 380 to 780 nm and *A* is Avogadro constant (6.022×10^23^*No*./*mol*). *E*_*B*_ was calculated based on the following assumption: Under normal growth condition without stress, 100 g choy sum edible portion (which had 94.2% of moisture content) contained 49 kJ of energy (Wills et al., 1984).

Based on the study by Sirtautas et al. (2014), NAR was modified and defined as the increase in dry weight (mg) of the whole choy sum seedling per unit leaf area (m^2^) per unit time (d) under certain light intensity and expressed as follows:

(7) $$\text{NAR}\,\left[\text{g}\,/\,\left({\text{m}}^{2}\cdot \text{d}\right)\right]=\frac{D\,W_{N}-D\,W_{0}}{T\,L\,A\cdot N},$$

Where *DW*_*N*_, *DW*_0_, and *N* are the same as defined in Section “Determination of Relative Growth Rate (μ)”. *TLA* is the total leaf area (m^2^) of choy sum seedling on day *N*.

Referred to Tichá et al. (1985), the calculation of *P*_*n*_ in this study was done by determining the increase in leaf dry weight (μg) per unit leaf area (m^2^) per unit time (s) of choy sum seedling under certain light intensity, and expressed as follows:

(8) $$P_{n}\,\left[\mu \,g\,/\,\left({\text{m}}^{2}\cdot \text{s}\right)\right]=\frac{L\,D\,W_{N}-L\,D\,W_{L}}{T\,L\,A\cdot \left(N-L\right)\cdot 43,200},$$

Where *LDW*_*N*_ and *LDW*_*L*_ stand for the total leaf dry weight (μg) of the whole choy sum seedling measured at the same timing on day *N* (when harvesting) and day *L* (when the first true leaf was shown) under certain light intensity, respectively, TLA is the total leaf area (m^2^) of the whole choy sum seedling under day *N* and under certain light intensity, “*N*” represents the duration of cultivation (days). “*L*” stands for the day when choy sum seedling just comes into the 1-leaf stage. “43,200” is the seconds of 12 h per day.

As a prediction tool, a 2D light response model can describe the relationship between *P*_*n*_ and light intensity, which may in-turn be used to predict *P*_*n*_ based on light intensity. A sigmoid-logistic model (SM) was employed for fitting the determined *P*_*n*_ data to obtain the best model that was judged by its highest coefficient of determination (*R*^2^) achieved as compared to other fitting formulae by using OriginPro 8.5.0 software, and expressed as follows:

(9) $$P_{n}=\frac{P_{m\,a\,x}}{1+e^{-k_{1}\,\left(I-I_{m/2}\right)}},$$

Where *P*_*max*_ [g/(m^2^⋅d)] stands for the maximal *P*_*n*_ that is achievable, *I*_*m/2*_ [μmol/(m^2^⋅s)] represents the light intensity to reach half of *P*_*max*_, *k*_1_ is a coefficient whose unit is (m^2^⋅s/μmol), *I* [μmol/(m^2^⋅s)] represents the light intensity applied.

### Selection of Optimal 2D Time-Based Growth Model and Construction and Selection of Optimal 3D Light-Time-Biomass Response Model

Selection of the optimal 2D time-based growth model was conducted by firstly choosing various growth-related models like Logistic, Gompertz and Exponential ones while setting linear model as the control (Table 2). The fitting of respectively designated models was then executed by employing DW data under Day 0 (DW of choy sum seed averaged as 0.5 mg/seed), 1-, 2-, and 3-leaf stages, as well as under Day 14 and Day 16 using OriginPro 8.5.0 software (Table 3). The goodness-of-fit of respective models was assessed by their *R*^2^ as calculated below:

(10) $$R^{2}=1-\frac{\text{RSS}}{\text{TSS}},$$

**TABLE 2:** The applied 2D and 3D models in this study.

Model category	Model name	General model formula/Formula in this study	^*a*^*RSS*	*F*-value	*p*-value (Prob > *F*)	Data points (*n*)	Parameter No. (*p*)	Region No.
2D	Linear (Control)	Db⁢W=D⁢W0+A⁢t/cD⁢WI-400=0.5+2.33⁢t	52.990	2.052	2.3 × 10^–1^	6	2	Inapplicable
2D	Logistic	D⁢W=A1+e-k⁢(t-tc)/(Shown⁢in⁢Table⁢ 4)	0.689	75007	8.9 × 10^–8^	6	3	Inapplicable
2D	Gompertz	*DW* = *Ae*^−*e*^[−*k*(*t*−*t*_*c*_)]^^/*DW*_*I*−400_ = 27,802*e*^−*e*^[−0.06(*t*−42.2)]^^	0.414	124684	4.2 × 10^–8^	6	3	Inapplicable
2D	Exponential	*DW* = *DW*_0_ + *Ae*^*R*_0_*t*^/*DW*_*I*−400_ = −1.3 + 1.81*e*^0.33*t*^	1.216	42481	2.1 × 10^–7^	6	3	Inapplicable
3D	Plane1 (Control)	*DW* = *DW*_0_ + *At* + *BI*(*A* > 0,*B* > 0)/	1590	134	0	56/6	3	1/2
		*DW* = −92.8 + 9.39*t* + 11.48*I*						
3D	LogisticCum	D⁢W=D⁢W0+B[1+eC-tD]⁢[1+eE-IF]/(Shown⁢in⁢Table⁢ 5)	32.638	4311	0	56/6	6	1/2
3D	Exponential2D	$D\,W=D\,W_{0}+B\,e^{\left(-\frac{t}{C}-\frac{I}{D}\right)}/D\,W=-9.5+1.27\,e^{\left(\frac{t}{4.55}+\frac{I}{4.17}\right)}$	173.892	1187	0	56/6	4	1/2
3D	GaussCum	$DW=DW_{0}+0.25B\left[1+\text{erf}\left(\frac{t-C}{\sqrt{2}\,D}\right)\right]\left[1+\text{erf}\left(\frac{I-E}{\sqrt{2}\,F}\right)\right]/$	31.307	4495	0	56/6	6	1/2
		$D\,W\,=1.9+116\,\left[1+\text{erf}\,\left(\frac{t-14.25}{5.01}\right)\right]\,\left[1+\text{erf}\,\left(\frac{I-5.25}{3.94}\right)\right]$						
3D	Plane2	*DW* = *DW*_0_ + *At* + *BI*(*A* > 0,*B* < 0)/(Shownin*Table* 5)	184.831	1113	1.9 × 10^–8^	6	3	2 only

*^*a*^*RSS*, residual sum of squares.*

*^*b*^*DW* represents dry weight of choy sum seedling (mg/seedling).*

*^*c*^*DW*_*I–400*_ stands for respective model formula under 400 μmol/(m^2^⋅s) in this study.*

**A*, *B*, *C*, *D*, *DW*_0_, *E*, *F, k, R_0_*, and *t*_*c*_ stand for the parameters in respective models. *I* represent light intensity [μmol/(m^2^⋅s)] and *t* is the cultivation duration (d). In this study, 56 of data points are applicable to model in Region 1, while 6 of data points are applicable to model in Region 2.*

**TABLE 3:** Dry weights of choy sum seedlings grown under different light intensities and growth stages.

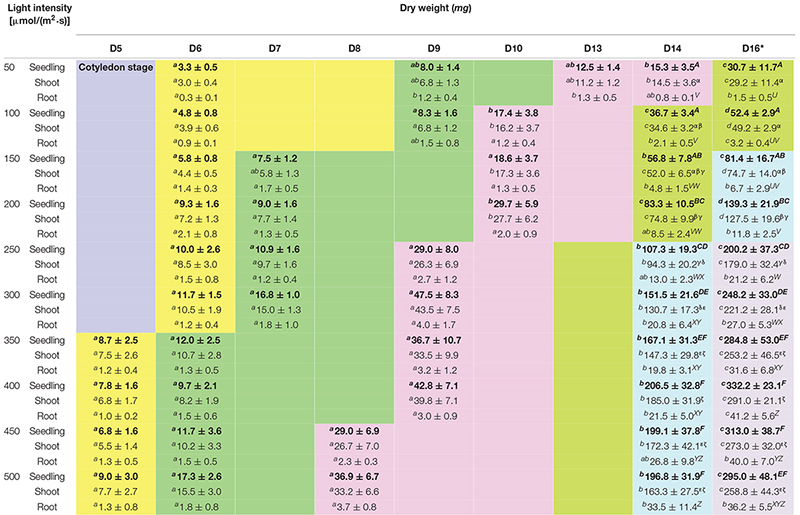

Data are expressed as means ± SD (*n* = 6). *Indicate that Day 16 is the transplantation day. Bold type data emphasize the dry weight value of the whole seedling under various light intensities tested. Table backgrounds in purple, yellow, light green, pink, olivine, blue, and lilac colors represented choy sum seedlings in their cotyledon, 1-, 2-, 3-, 4-, 5-,and 6-leaf stages, respectively. Different lower cases (a, b, c, and d) show remarkable differences among different growth stages under the same light intensity. Different upper cases (A, B, C, D, E, and F), Greek alphabet (α, β, γ, δ, ε, and ζ) and upper cases (U, V, W, X, Y, and Z) indicate significant differences among various light intensities tested [one-way analysis of variance (ANOVA); Tukey multiple comparison; *p* < 0.05].

Where RSS is the residual sum of squares and TSS represents the total sum of squares (Ghate et al., 2016).

The overfitting evaluation of the models was determined by comparing the Akaike Information Criterion (AIC) values of respective models. The AIC value is defined as follows:

(11) $$\text{AIC}=\text{n}\cdot \ln\left(\frac{\text{RSS}}{n}\right)+2\,p,$$

Where *n* is the number of experimental data points and *p* stands for the total number of parameters included in respective models applied (Table 2) (Bolboacã and Jäntschi, 2009).

**FIGURE 2:**
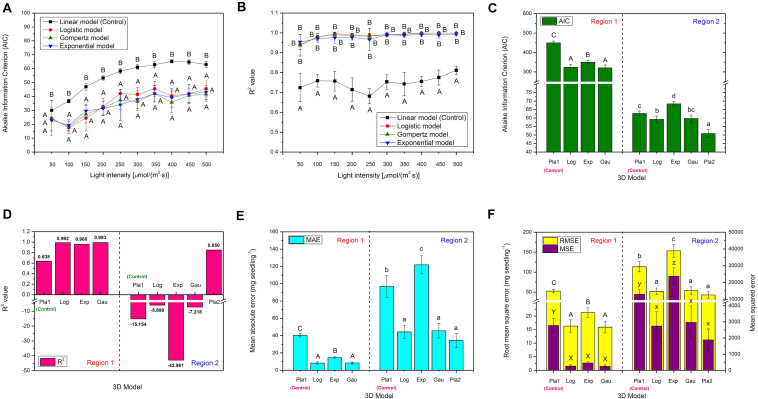
The comparison of various 2D time-based growth models based on **(A)** Akaike Information Criterion (AIC) value and **(B)** *R*^2^ value of choy sum seedling grown under different white LED light intensities, as well as the comparison of various 3D light-time-biomass response models based on **(C)** AIC value, **(D)** *R*^2^ value, **(E)** Mean absolute error (MAE), and **(F)** Root mean square error (RMSE) and Mean squared error (MSE). Pla1, Plane1 model; Log, LogisticCum model; Exp, Exponential2D model; Gau, GaussCum model; Pla2, Plane2 model. Different upper cases (A, B, C, X, and Y) and lower cases (a, b, c, x, y, and z) indicate significant differences among all the 2D and 3D models tested [*n* = 6, one-way analysis of variance (ANOVA); Tukey multiple comparison; *p* < 0.05].

High *R*^2^ value and low AIC value were the criteria for selecting the optimal 2D time-based growth model (Figures 2A,B). Then, the 3D light-time-biomass response model was built on the selected 2D time-based growth models of choy sum seedling under different light intensities. The light-time-based DW data were then arranged into the form of a matrix and subsequently fitted to various 3D models including LogisticCum, Exponential2D, GaussCum, and Plane2 (in which the duration variable is positively correlated but the light intensity variable is negatively correlated to the growth), along with Plane1 (in which the duration and light intensity variables are both positively correlated to the growth) as the control, and analyzed by OriginPro 8.5.0 software. The selection criteria for optimal 3D light-time-biomass response model were the same as those for the 2D model, i.e., high *R*^2^ value and low AIC value (Figures 2C,D).

### Structure of the Final 2D Time-Based Growth Model and 3D Light-Time-Biomass Response Model

The finally selected 2D time-based growth model was the logistic model (Table 4) and is expressed as follows:

(12) $$D\,W_{I}=\frac{D\,W_{m\,a\,x}}{1+e^{-k_{2}\,\left(t-t_{m/2}\right)}},$$

**TABLE 4:** The selected 2D light response model and 2D time-based (logistic) growth model of choy sum seedling.

Model	Condition	Coefficient		^*a*^*SD*	Model formula	^*b*^*Adj. R^2^*	^*c*^*RMSE*
2D light response model	Day 14	*P*_*max*_	124.7	7.2	d⁢Pn⁢(D⁢a⁢y⁢ 14)=124.71+e-0.007⁢(I-101.3)	0.963	0.4
		*I*_*m/2*_	101.3	14.2			
		*k*_1_	0.007	0.002			
	Day 16	*P*_*max*_	155.9	19.4	Pn⁢(D⁢a⁢y⁢ 16)=155.91+e-0.005⁢(I-329.2)	0.992	0.3
		*I*_*m/2*_	329.2	55.4			
		*k*_1_	0.005	0.001			
2D time-based growth model	50 μmol/(m^2^⋅s)	^*e*^*DW*_*max*_	18.1	3.7	$D\,W_{I-50}=\frac{18.1}{1+e^{-0.35\,\left(t-10.2\right)}}$	0.975	6.0
		*t*_*m/2*_	10.2	1.4			
		*k*_2_	0.35	0.04			
	100 μmol/(m^2^⋅s)	*DW*_*max*_	90.3	25.2	$D\,W_{I-100}=\frac{90.3}{1+e^{-0.34\,\left(t-15.1\right)}}$	0.988	1.8
		*t*_*m/2*_	15.1	1.7			
		*k*_2_	0.34	0.03			
	150 μmol/(m^2^⋅s)	*DW*_*max*_	109.4	20.5	$D\,W_{I-150}=\frac{109.4}{1+e^{-0.39\,\left(t-13.7\right)}}$	0.990	1.9
		*t*_*m/2*_	13.7	0.9			
		*k*_2_	0.39	0.02			
	200 μmol/(m^2^⋅s)	*DW*_*max*_	182.4	37.5	$D\,W_{I-200}=\frac{182.4}{1+e^{-0.42\,\left(t-14.1\right)}}$	0.987	6.2
		*t*_*m/2*_	14.1	0.9			
		*k*_2_	0.42	0.02			
	250 μmol/(m^2^⋅s)	*DW*_*max*_	232.9	67.7	$D\,W_{I-250}=\frac{232.9}{1+e^{-0.44\,\left(t-13.7\right)}}$	0.965	13.9
		*t*_*m/2*_	13.7	1.2			
		*k*_2_	0.44	0.03			
	300 μmol/(m^2^⋅s)	*DW*_*max*_	258.7	36.5	$D\,W_{I-300}=\frac{258.7}{1+e^{-0.5\,\left(t-12.3\right)}}$	0.986	16.1
		*t*_*m/2*_	12.3	0.6			
		*k*_2_	0.50	0.03			
	350 μmol/(m^2^⋅s)	*DW*_*max*_	280.5	63.5	$D\,W_{I-350}=\frac{280.5}{1+e^{-0.5\,\left(t-12.5\right)}}$	0.952	21.1
		*t*_*m/2*_	12.5	1.0			
		*k*_2_	0.50	0.04			
	400 μmol/(m^2^⋅s)	*DW*_*max*_	419.5	48.9	$D\,W_{I-400}=\frac{419.5}{1+e^{-0.49\,\left(t-13.5\right)}}$	0.989	12.3
		*t*_*m/2*_	13.5	0.6			
		*k*_2_	0.49	0.02			
	450 μmol/(m^2^⋅s)	*DW*_*max*_	355.8	38.3	$D\,W_{I-450}=\frac{355.8}{1+e^{-0.51\,\left(t-12.9\right)}}$	0.988	13.8
		*t*_*m/2*_	12.9	0.6			
		*k*_2_	0.51	0.02			
	500 μmol/(m^2^⋅s)	*DW*_*max*_	262.0	37.3	$D\,W_{I-500}=\frac{262.0}{1+e^{-0.56\,\left(t-11.0\right)}}$	0.968	22.0
		*t*_*m/2*_	11.0	0.6			
		*k*_2_	0.56	0.03			

*^*a*^*SD*, standard deviation.*

*^*b*^*Adj*., adjusted.*

*^*c*^*RMSE*, root mean square error.*

*^*d*^*P*_*n*_, net photosynthetic rate.*

*^*e*^*DW*, dry weight. *RMSE* unit for 2D light response model is “μg/(m^2^⋅s)”. *RMSE* unit for 2D time-based growth model is “mg/seedling.”*

Where *DW*_*I*_ and *DW*_*max*_ are the actual and maximal dry weights achievable (mg) of whole choy sum seedling under a given light intensity, *t*_*m/2*_ (d) stands for the time required to reach half of *DW*_*max*_, *k*_2_ is a coefficient whose unit is d^–1^, *t* represents the cultivation duration (d).

The 3D light-time-biomass response model is a new concept proposed in this study, which aids in predicting the biomass of choy sum seedling on a dry mass basis, under any cultivation time and any light intensity within the range set in the study [i.e., Day 0 to Day 16 of duration and 50–500 μmol/(m^2^⋅s) of light intensity]. Due to the influence of light saturation point, two different 3D models were adopted for the two distinctive sets of duration and light intensity conditions (designated as Region 1 and 2). In Region 1, the finally selected nonlinear light-time-biomass response (LogisticCum) model is expressed as follows:

(13) $$D\,W_{t-I}\,=D\,W_{0}+\frac{D\,W_{m\,a\,x}}{\left[1+e^{\frac{t_{m/4}-t}{k_{3}}}\right]\,\left[1+e^{\frac{I_{m/4}-I}{k_{4}}}\right]},$$

Where *DW*_*t–I*_ stands for DW (mg) of the whole choy sum seedling under a particular time and light intensity, *DW*_0_ (mg) represents the initial average mass of choy sum seed portion that will be developed into the seedling, *DW*_*max*_ (mg) is the maximal DW achievable in the germination tray, *t*_*m/4*_ (d) and *I*_*m/4*_ [μmol/(m^2^⋅s)] are the time and the light intensity, respectively, to achieve 25% of *DW*_*max*_ when being combined in application. *k*_3_ (d) and *k*_4_ [μmol/(m^2^⋅s)] are coefficients, *t* represents the cultivation duration (d) and *I* stands for the light intensity [μmol/(m^2^⋅s)] applied. The nonlinear light-time-biomass response (logisticCum) model in (13) is applicable to seedling grown below 400 μmol/(m^2^⋅s) or below 14 days of cultivation duration, i.e., Region 1. In Region 2, the finally selected light-time-biomass response (plane) model is as below:

(14) $$DW_{t-I}=DW_{c}+k_{5}t+k_{6}I\left(k_{5}>0,k_{6}<0\right),$$

Where *DW*_*t–I*_ stands for DW (mg) of the whole choy sum seedling under a particular time longer than 14 days till 16 days and light intensity higher than 400 μmol/(m^2^⋅s) till 500 μmol/(m^2^⋅s), *DW*_*c*_ (mg) is a derived value from DW under 400 μmol/(m^2^⋅s) on Day 14, *k*_5_ (mg/d) and *k*_6_ (mg⋅m^2^⋅s/μmol) are coefficients corresponding to the factors of time and intensity, respectively. This light-time-biomass response (plane) model in (14) is applicable to seedling grown above the light saturation point. Details of the variable ranges for the 3D models in Regions 1 and 2 are shown in Table 5.

**TABLE 5:** The selected 3D light-time-biomass response model of choy sum seedling.

Model	Variable range	Region	Coefficient		^*a*^*SD*	Model formula (mg/seedling)
3D light-time-biomass response model	(1) *t* ϵ day [0–16] and *I* ϵ [50–400] μmol/(m^2^⋅s) (2) *t* ϵ day [0–14) and *I* ϵ [50–500] μmol/(m^2^⋅s)	1	^*b*^*DW*_0_	0.8	0.4	$D\,W_{t-I}=0.8+\frac{441.7}{\left[1+e^{\frac{13.02-t}{2.05}}\right]\,\left[1+e^{\frac{259.5-I}{83.5}}\right]}$
			^*c*^*DW*_*max*_	441.7	18.5	
			*t*_*m/4*_	13.02	0.15	
			*k*_3_	2.05	0.08	
			*I*_*m/4*_	259.5	5.50	
			*k*_4_	83.5	3.00	
	(3) *t* ϵ day [14–16] and *I* ϵ (400–500] μmol/(m^2^⋅s)	2	^*d*^*DW*_*c*_	10.6	0.7	*DW*_*t*−*I*_ = 10.6 + 30.47*t*−0.46*I*
			*k*_5_	30.47	5.55	
			*k*_6_	-0.46	0.09	

*^*a*^*SD*, standard deviation.*

*^*b*^*DW*_0_, dry weight of choy sum seed portion that will be developed into the seedling.*

*^*c*^*DW*_*max*_, the maximal dry weight achieved.*

*^*d*^*DW*_*c*_, the coordinated dry weight in Region 2.*

### Robustness Test of 3D Light-Time-Biomass Response Model

The robustness test of 3D light-time-biomass response model of choy sum seedling was carried out by using the model variation test (Neumayer and Plümper, 2017), which could be achieved by substituting the regressor such as light intensity *I* [μmol/(m^2^⋅s)] in Formula (13) by light energy *E* [J/(m^2^⋅s)] as Formula (15) in Region 1:

(15) $$D\,W_{t-I}=D\,W_{0}+\frac{D\,W_{m\,a\,x}}{\left[1+e^{\frac{t_{m/4}-t}{k_{3}}}\right]\,\left[1+e^{\frac{E_{m/4}-E}{k_{4}}}\right]},$$

as well as replacing light intensity *I* [μmol/(m^2^⋅s)] in Formula (14) by light energy *E* [J/(m^2^⋅s)] as Formula (16) in Region 2:

(16) $$DW_{t-I}=DW_{c}+k_{5}t+k_{6}E\left(k_{5}>0,k_{6}<0\right),$$

and subsequently analyzing the *t*-values of all the parameters involved such as *DW*_0_, *DW*_*max*_, *t*_*m/4*_, *k*_3_, *E*_*m/4*_, and *k*_4_ in Region 1 and *DW*_*c*_, *k*_5_, and *k*_6_ in Region 2 to check whether their Prob > | *t*| are also significant (*p* < 0.05), similar to those of the 3D light-time-biomass response model, if yes, then this 3D model could be considered robust enough.

### Statistical Analysis

The experiments studying morphological and growth parameters, RGR, productivity, PE, NAR, and *P*_*n*_ of choy sum seedling were performed in sextuplicate (*n* = 6). The results were analyzed by one-way analysis of variance (ANOVA) using Tukey’s multiple comparison to evaluate the differences among different growth stages under the same light intensity, and among all the light intensities tested or through student’s *t*-test to estimate the differences between two independent conditions. Differences were considered significant at *p* < 0.05. All statistics were performed by the software of Statistical Product and Service Solutions (SPSS Version 17.0).

## Results and Discussion

### Effect of Light Intensity on the Environmental Conditions of Choy Sum Living

**FIGURE 3:**
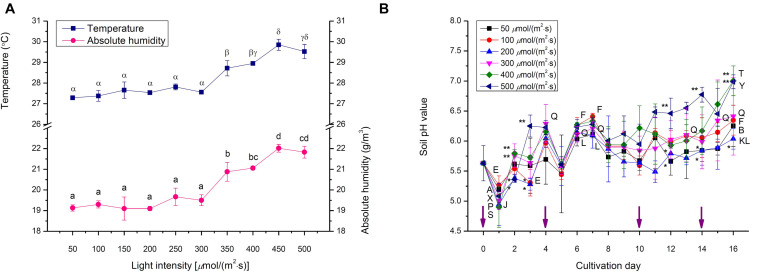
The environments of **(A)** temperature and absolute humidity, as well as **(B)** soil pH of choy sum seedlings grown under different white LED light intensities and days. Different Greek alphabets (α, β, γ, and δ) and lower cases (a, b, c, and d) showed significant differences of temperature and absolute humidity across all the light intensities tested, respectively. Different upper cases (A, B/E, F/J, K, L/P, Q/S, T/X, and Y) indicated significant differences of soil pH values among 50, 100, 200, 300, 400, and 500 μmol/(m^2^⋅s), respectively. The asterisks (* and **) indicated significant differences of soil pH values under specific light intensities on the identical day (*n* = 3, one-way ANOVA; Tukey multiple comparison; *p* < 0.05). The purple arrows indicated the irrigation days.

In this work, the environmental conditions of choy sum living, such as temperature, absolute humidity and soil pH value, were evaluated (Figure 3). Temperature from 50 to 300 μmol/(m^2^⋅s) demonstrated no significant difference, while its remarkable increase from 27.6 to 29.9°C was found when light intensity was increased from 300 to 500 μmol/(m^2^⋅s). The variation tendency of absolute humidity was the same as that of temperature. A significant increase from 19.5 to 22.0 g/m^3^ was evident when the light intensity increased from 300 to 500 μmol/(m^2^⋅s). Higher light intensities tended to result in higher absolute humidity (Figure 3A), which could be due to the stronger transpiration effect of seedling generated under a higher light intensity environment. Higher light intensities help to develop bigger shoot with more surface area exposed to the environment, through which transpiration is strengthened. In addition, they also boost the development of robust choy sum root systems (Figures 1D,E) for accelerating the absorption of ions/minerals from the soil into the root cells, thus increasing soil water potentials, which showed a positive relation with transpiration (Razzaghi et al., 2011).

The initial pH value of soil containing black peat as the main component was 5.63 (Figure 3B). In this case, soil pH values under different light intensities were found significantly elevated with time elapsing by up to 7.00 on day 16, which could be the selective absorption of certain kinds of soluble NPK nutrients and minerals in soil by choy sum root, resulting that large amounts of OH^–^ originally inside the root cells were substituted and released into the soil, leading to soil pH increase (Haynes, 1980). Interestingly, the application of water for solving more of NPK nutrients and minerals from the soil could be the reason that led to the drop of soil pH value one day after the irrigation (i.e., on day 1 and 5 at the early stage). Nevertheless, such declining significances were not found at the late stage on day 11 and 15, respectively, revealing that the soluble NPK nutrients and minerals could be completely solved from the soil at this stage. Furthermore, soil pH under higher light intensities demonstrated higher values, indicating that choy sum seedling under higher light intensities received more light energy to facilitate its absorption of more of anions from soil NPK salts through selective absorption property, thus increase pH of substrate, compared with those under lower light ones (Haynes, 1980) (Figure 3B). The soil pH value of the current work on choy sum seedling was similar to the previous study to cultivate Chinese cabbage (*Brassica rapa* ssp. *campestris*) in the greenhouse (pH 6.2) and outdoor (pH 7.6), revealing that ion compositions in this commercial substrate could match the growth of choy sum seedling in this study (Lee et al., 2010).

### Effect of Light Intensity on the Morphology of Choy Sum

**FIGURE 4:**
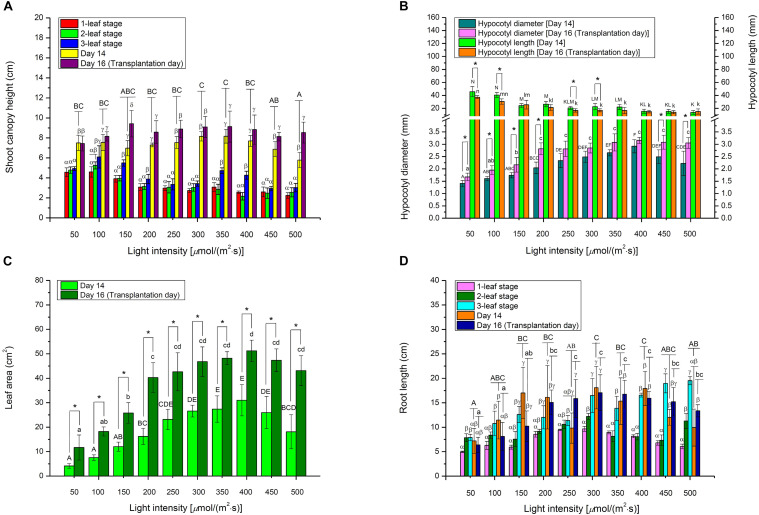
The morphological parameters, including **(A)** shoot canopy height, **(B)** hypocotyl diameter and length, **(C)** leaf area, and **(D)** root length, of choy sum seedling grown under different white LED light intensities and growth stages. Different Greek alphabets (α, β, γ, and δ) showed significant differences among different growth stages as well as Day 14 and Day 16 under the same light intensity. Different upper cases (A, B, C, D, E, F, K, L, M, and N) and lower cases (a, b, c, d, k, l, m, and n) indicated significant differences across all the light intensities tested on Day 14 and Day 16, respectively (*n* = 6, one-way ANOVA; Tukey multiple comparison; *p* < 0.05). The asterisk (*) indicates significant differences between Day 14 and Day 16 within each light intensity (*n* = 6, Student’s *t* test, *p* < 0.05).

At the early 1- and 2-leaf stages, choy sum shoots grown under below 100 μmol/(m^2^⋅s) demonstrated up to a 2.03-fold increase in canopy height compared with those under higher light intensities. This was because low light intensity led to etiolation, where stems grew longer toward higher light direction (Figures 1D,E, 4A). These results were in agreement with those for another *Brassica* species (*Brassica napus* L. cv. Westar), whose seedling height grown under 25 μmol/(m^2^⋅s) was 4.5-fold higher than those under 500 μmol/(m^2^⋅s) at the early stage (Day 7) (Potter et al., 1999). Yet on Day 16, no significant difference in canopy heights among the shoots was found although hypocotyl lengths were progressively decreased with increasing light intensity (Figures 1D,E, 4A,B), showing that shoot canopy heights under various light intensities at the late seedling stage were not markedly affected by hypocotyl length and tended to be the same.

Under a high light intensity, a short stem structure, contributing to improve the lodging resistance of plants, may be formed (Zhang et al., 2014), which also occurred to choy sum (Figures 1D,E), shorter choy sum hypocotyl length thus formed may be explained by the regulation of blue-light-induced cryptochrome 1 protein, a blue light sensing photoreceptor. Blue light may up-regulate the expression of *Brassica napus* cryptochrome 1 gene (*BnCRY1*) that are capable of controlling the plant’s hypocotyl/stem to grow shorter (Chatterjee et al., 2006). In this study, higher light intensity also contained higher amount of blue light, the high blue light environment was deemed to trigger the action of *BnCRY1* to inhibit the hypocotyl’s elongation accordingly (Table 1 and Figure 4B). These results were also in agreement with some *Brassica* microgreens such as Mustard (*B. juncea* L.), Tatsoi (*B. rapa var. rosularis*) and Kohlrabi (*B. oleracea var. gongylodes*), as well as *B. napus*, for which decreased hypocotyl lengths were negatively related to increased light intensity (Potter et al., 1999; Samoulienë et al., 2013). Based on Figures 4B,C and Table 1, bigger hypocotyl diameter and shorter hypocotyl length, induced by higher light intensity, could support larger leaf area, and the synchronous development in both stem and leaf was strongly related to light intensity. The trend observed for hypocotyl diameter was similar to that reported for cherry tomato plant (*Solanum lycopersicum Mill qianxi*.), where stem diameter was also positively correlated to light intensity (Fan et al., 2013).

Choy sum total leaf areas measured on Day 14 grown under 350 and 400 μmol/(m^2^⋅s), as well as on Day 16 under 400 μmol/(m^2^⋅s), were found to be significantly higher than those grown under lower [<250 μmol/(m^2^⋅s)] or higher light [>450 μmol/(m^2^⋅s)] intensities. This result was in line with those of an earlier study which reported markedly bigger leaf areas for some *Brassica* microgreens grown between 330 and 440 μmol/(m^2^⋅s), compared to those grown under 110, 220, and 545 μmol/(m^2^⋅s) (Figure 4C) (Samoulienë et al., 2013). Both results seemed to indicate that light intensities between 330 and 450 μmol/(m^2^⋅s) were the optimal range for the growth of some *Brassica* species while higher light intensity may give rise to photo-inhibition. Similarly, other *Brassica* species such as *Brassica fruticulosa* and *Brassica oleracea* have been shown to have significantly smaller leaf areas when cultivated in excessively high light intensities under 1200 μmol/(m^2^⋅s), compared to the control [60 μmol/(m^2^⋅s)] (Díaz et al., 2007). Under extremely high illumination, photosystem II (PSII) quantum yield and photosynthetic electron transport capacity were reduced, whereas photosystem I (PSI) activity was increased. The expression of thylakoidal NADH dehydrogenase complex (NADH-DH) in thylakoid membranes, and plastid terminal oxidase (PTOX) in plastoquinone oxidation involved in chlororespiration process, were also increased. The activation of the chlororespiration process improved the adaptation of *Brassica* plants to extremely high illumination and heat, but led to leaf area reduction in *Brassica* species (Díaz et al., 2007).

In this study, root length of choy sum showed a significantly increasing trend when light intensity was enhanced and higher growth stage was achieved (Figures 1D,E, 4D). Another study using *Brassica* species termed mini Chinese cabbage (*Brassica pekinensis* cv. “Jinwa no. 2”) also reported similar results, whereby its root length treated below 100 μmol/(m^2^⋅s) was markedly shorter than that treated by 200 μmol/(m^2^⋅s) (Hu et al., 2015). Roots are responsible for absorbing water and nutrients from soil for seedling’s healthy growth, hence increasing root system length would be more conducive to improving crop quality. However, root system development is regulated by auxin. A small concentration of as low as 10^–10^ M is required for optimal development, whereas a higher concentration may suppress root elongation and promote adventitious root formation instead (Shaheed, 2017). Several auxin-inducible genes like the primary auxin response gene IAA3, were up-regulated in the wild-type *Brassicaceae* species *Arabidopsis* as a response to decreased light intensity, revealing that more auxins may be synthesized under low intensity, which would then inhibit root elongation, as showed by our study (Figure 4D) (Vandenbussche et al., 2003).

### Effects of Light Intensity on the Growth of Choy Sum Seedling

**FIGURE 5:**
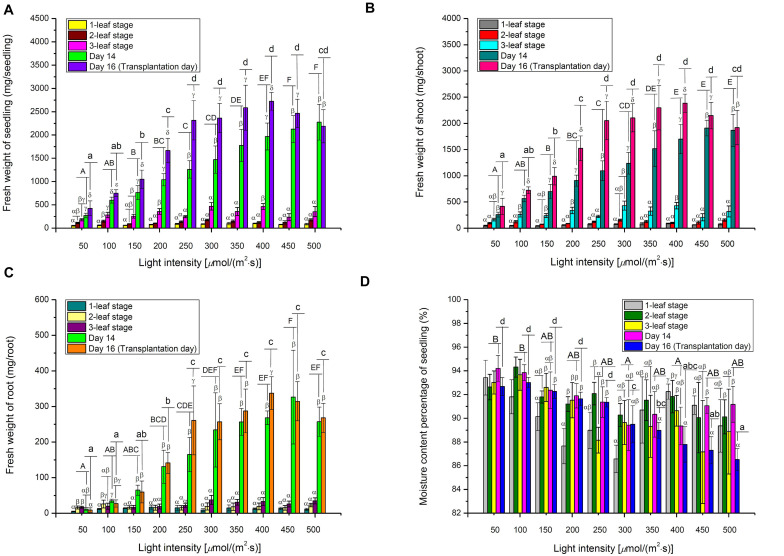
The fresh weights of **(A)** Seedling, **(B)** Shoot, **(C)** Root, as well as **(D)** Moisture content of choy sum seedlings grown under different white LED light intensities and growth stages, as well as Day 14 and Day 16. Different Greek alphabets (α, β, γ, and δ) indicate significant differences among different growth stages, Day 14 and Day 16 under the same light intensity. Different upper cases (A, B, C, D, E, and F) and lower cases (a, b, c, and d) indicated significant differences among all the light intensities tested on Day 14 and Day 16, respectively (*n* = 6, one-way ANOVA; Tukey multiple comparison; *p* < 0.05).

**FIGURE 6:**
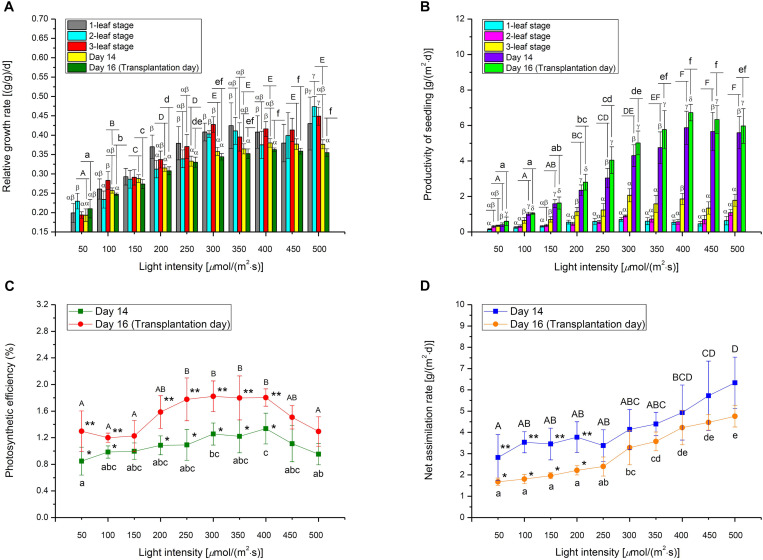
**(A)** Relative growth rate, **(B)** Productivity, **(C)** Photosynthetic efficiency, and **(D)** Net assimilation rate of choy sum seedling grown under different white LED light intensities and growth stages and/or Day 14 and Day 16. Different Greek alphabets (α, β, γ, and δ) show significant differences among different growth stages, Day 14 and Day 16 under the same light intensity. Different upper cases (A, B, C, D, E, and F) and lower cases (a, b, c, d, e, and f) indicated significant differences among all the light intensities tested on Day 14 and Day 16, respectively (*n* = 6, one-way ANOVA; Tukey multiple comparison; *p* < 0.05). The asterisks (* and **) indicate significant differences between Day 14 and Day 16 (*n* = 6, Student’s *t* test, *p* < 0.05).

On Day 14, FWs of choy sum seedling and shoot demonstrated an almost linear increase with the increase in light intensity, achieving as much as 8.40- and 7.37-fold of increase, respectively, at 500 μmol/(m^2^⋅s) as compared to 50 μmol/(m^2^⋅s) (Figures 5A,B). The results suggested that a 10-fold increase in light energy input may not generate an equivalent 10-fold increase in choy sum FW. However, seedling DW grown under 400 μmol/(m^2^⋅s) was 13.50 and 2.48 times greater than those grown under 50 and 200 μmol/(m^2^⋅s) (the control) on Day 14, respectively (Table 3). Hence, an eightfold increase in light energy input induced a comparatively larger 13.47-fold increase in dry mass output. This was consistent with the results of RGR (μ), where the seedlings grown under above 300 μmol/(m^2^⋅s) displayed a higher RGR of up to 1.96 times compared with those grown under lower light intensities (Figure 6A). Intriguingly, FWs of choy sum seedling, shoot and root on Day 16 displayed an ascending trend only below 250 μmol/(m^2^⋅s). For example, their values from 50 to 200 μmol/(m^2^⋅s) were progressively increased and those from 250 to 500 μmol/(m^2^⋅s), although becoming significantly higher, were close to each other, revealing that light intensity within a certain range may achieve a similar result in choy sum FW (Figures 5A–C). A comparison on root system development under all the light intensities studied was given in Figure 1, from which choy sum root system was slowly improved with increasing light intensity. Meanwhile, their fresh and dry masses were markedly increased (Table 3 and Figure 5C).

As found, DW of *Brassica napus* L. cv. Westar seedlings harvested after 17-days of cultivation under 500 μmol/(m^2^⋅s) was 240 mg, which was lower than our current 295.0 mg on Day 16 under the same light intensity. In addition, *B. napus* seedlings grown under 500 μmol/(m^2^⋅s) were 1.71 times greater in DW as compared to those grown under 250 μmol/(m^2^⋅s) (Potter et al., 1999). Similarly, a 1.47-fold increase of choy sum seedling on Day 16 was observed under the same lighting conditions (Table 3), indicating that dry mass accumulation of some *Brassica* species under a progressively increasing light intensity may have similar growth trends. Other *Brassica* species, like Red Pak Choi, Tatsoi and Kohlrabi, also demonstrated a similar trend (Samoulienë et al., 2013).

High light intensity accelerated true leaf development in choy sum seedlings. Seedlings grown under above 350 μmol/(m^2^⋅s) entered the 1-leaf stage on Day 5, while those grown under below 300 μmol/(m^2^⋅s) were still in the cotyledon stage. On Day 6, seedlings grown under below 300 μmol/(m^2^⋅s) entered the 1-leaf stage, whereas those grown under above 350 μmol/(m^2^⋅s) already entered the 2-leaf stage. In addition, seedlings treated at above 450 μmol/(m^2^⋅s) reached the 3-leaf stage on Day 8, which was one day ahead of those grown under between 250 and 400 μmol/(m^2^⋅s). In contrast, the development of seedlings grown under lower light intensities was slower. Seedlings grown under below 200 μmol/(m^2^⋅s) entered the 3-leaf stage on Day 10, while those grown at 50 μmol/(m^2^⋅s) reached the 3-leaf stage as late as Day 13. By Day 16, seedlings grown under below 100 μmol/(m^2^⋅s), between 150 and 200 μmol/(m^2^⋅s), and above 250 μmol/(m^2^⋅s) had reached the 4-, 5-, and 6-leaf stages, respectively (Table 3). These results were in line with the above ornamental shrub study, where the true leave numbers emerged per unshaded plant was more than those grown under mildly and heavily shaded environments (Fini et al., 2010). Similar to the variation trend of true leaf development under different light intensities, choy sum seedling DW under higher light intensities at different growth stages showed higher values earlier, compared with those under lower light ones (Table 3).

**FIGURE 7:**
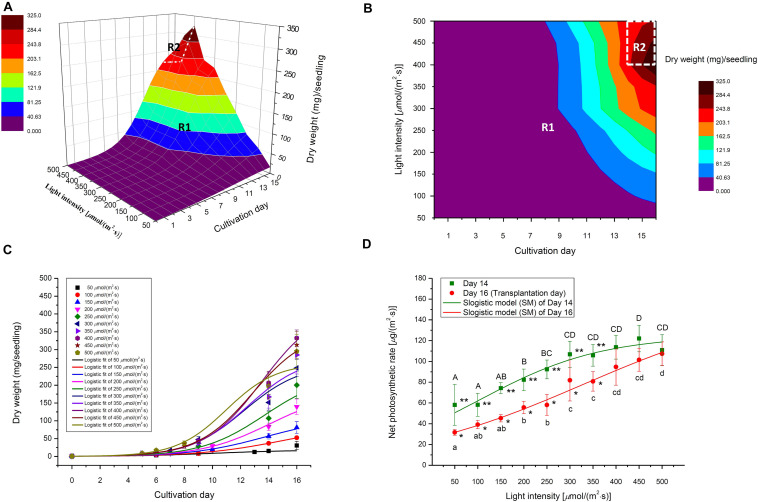
**(A)** Selected 3D light-time-biomass response model, **(B)** the light-intensity-and-time- based dry weight contour of selected 3D model, **(C)** selected 2D time-based growth model, and **(D)** Net photosynthetic rate (2D light response model) of choy sum seedling grown under different white LED light intensities and duration. R1 and R2 stand for Region 1 and Region 2 of the selected 3D light-time-biomass response model, respectively. Different upper cases (A, B, C, D) and lower cases (a, b, c, d) indicate significant differences among all the light intensities tested on Day 14 and Day 16, respectively (*n* = 6, one-way ANOVA; Tukey multiple comparison; *p* < 0.05). The asterisks (* and **) indicated significant differences between Day 14 and Day 16 (*n* = 6, Student’s *t* test, *p* < 0.05).

The significant decrease of moisture content in choy sum seedlings, with increasing light intensity, was mainly found on Day 16 (Figure 5D). This could be due to the higher heat load which higher far red light intensities (701–780 nm) imposed upon the seedlings, leading to a higher transpiration rate and hence lower moisture content in each seedling (Table 1). This relationship between light intensity and transpiration rate can also be explained by the 2D light response model (*P*_*n*_) described below. As known, photosynthetic rate of higher plants, such as wheat (*Triticum aestivum* L.), is usually positively correlated with stomatal conductance and yield, while stomatal conductance is also positively correlated with transpiration rate (Fischer et al., 1998; Tuzet et al., 2003). Taken altogether, these studies suggest that a high *P*_*n*_ achieved under high light would result in increased stomatal conductance and transpiration rate, eventually leading to low overall moisture content in choy sum seedlings at the time of transplantation (Figures 5D, 7D).

Choy sum seedling productivity is limited by the average seedling dry mass obtained and planting density. In this study, 1 m^2^ of germination tray contained up to 325 seedlings based on 0.0025 m^2^ (0.05 m × 0.05 m) of planting area for each seedling. The highest productivity in terms of DW achieved on Day 14 was 5.87 g/(m^2^⋅d) for plants grown under 400 μmol/(m^2^⋅s), compared to 6.73 g/(m^2^⋅d) on Day 16 under the same light intensity. Within each Day 14 or Day 16, the seedling productivity at above 400 μmol/(m^2^⋅s) showed no statistical difference, suggesting that stable choy sum seedling productivity can be maintained when sufficient light energy is applied (Figure 6B).

### Effects of Light Intensity on the Photosynthetic Parameters of Choy Sum Seedling

Photosynthetic efficiency (energy-related), *NAR* (seedling-biomass-related), and *P*_*n*_ (leaf-biomass-related) are the key photosynthetic parameters and the relationships between them and light intensity were shown in Figures 6C,D, 7D, respectively. *PE* can be expressed as the percentage of light energy received by the plant that is converted to chemical energy stored in plant biomass (Huang et al., 2016). Typically, up to 9.4% of solar PAR is eventually converted to above-ground biomass in C3 plants due to reflection and transmission losses, photochemical inefficiency, thermo-dynamic limit, energy losses by carbohydrate biosynthesis, photorespiration, and respiration (Zhu et al., 2010). Current results showed that a maximum *PE* of 1.34% was achieved for choy sum shoot part under 400 μmol/(m^2^⋅s) on Day 14, and 1.78–1.82% for those treated from 250 to 400 μmol/(m^2^⋅s) on Day 16, respectively (Figure 6C). Compared with all the other light intensities studied within the same day, choy sum shoot part under 250–400 μmol/(m^2^⋅s) was capable of converting a higher proportion of received light into chemical potential energy. Similarly, 300 μmol/(m^2^⋅s) gave rise to the highest energy efficiency (g/kW, in which plant mass unit was not transformed into energy unit) for cherry tomato (Fan et al., 2013). However, the above *PE* values for choy sum shoot part, while transplanting, are less than the early mentioned theoretical maximum *PE* of 9.4%, this is because such *PE* was calculated based on those mature crop plants with higher biomass (Zhu et al., 2010), hence it is still possible for choy sum shoot part to achieve a higher *PE* if the illumination condition such as the combination of light intensity, red blue light ratio and photoperiod is optimized at the seedling stage and subsequently growing it to the mature plant (Figure 6C). In this study, 350–450 μmol/(m^2^⋅s) was recognized as the optimal light intensity range due to the highest average *PE* and the highest average dry mass, which were significantly higher than those grown under 200 μmol/(m^2^⋅s) (the control) (Table 3). This range for the growth of choy sum seedling was compatible with the conclusion of Samoulienë et al. (2013) who showed that the optimal illumination condition for *Brassica* microgreens’ growth was 330–440 μmol/(m^2^⋅s).

In this study, *NAR* was used to measure choy sum growth (change in seedling DW per unit time) based on unit leaf area under different light intensities and it was found to be positively related to light intensity (Figure 6D) (Sirtautas et al., 2014). In addition, *NAR* values on Day 14 for seedlings grown under below 250 μmol/(m^2^⋅s) were found to be significantly higher than those on Day 16, while *NAR* values for seedlings grown under above 250 μmol/(m^2^⋅s) on Day 14 and Day 16 did not show any significant difference among them. This revealed that under a low light intensity, a shorter cultivation time may result in a higher *NAR*. The above findings implied that choy sum leaves carry out photosynthesis more efficiently either under high light intensity or under specific combinations of low light intensity and cultivation time. *NAR* values obtained in this study (Figure 6D) were close to a previously reported value of around 4 g/(m^2^⋅d) for tropical oil crop *Brassica campestris* L. var. Kranti (Singh et al., 2009).

The 2D light response model describes the relationship between *P*_*n*_ and light intensity and can be used to predict the increase in leaf dry mass accumulation per unit leaf area per unit time, under various light intensities. In this study, a SM model was applied to *P*_*n*_ data to obtain the best model structure and the corresponding model coefficients (Figure 7D and Table 4). The SM models under Day 14 and Day 16 fit well to the experimental data as indicated by both high *R*^2^ and low RMSE. Furthermore, the extension of cultivating duration from Day 14 to Day 16 was found to produce a higher *P*_*max*_, which also required a higher light intensity [329.2 μmol/(m^2^⋅s) on Day 16 versus 101.3 μmol/(m^2^⋅s) on Day 14] to achieve half of *P*_*max*_ (Table 4).

### 2D Time-Based Growth Model and 3D Light-Time-Biomass Response Model of Choy Sum Seedling

Selecting an appropriate model is the basis for reliably predicting biomass production. *R*^2^ and AIC are the two essential parameters to evaluate the fit goodness and overfitting of models. A high-quality model is characterized by low AIC and high *R*^2^. Looking at AIC values of various 2D models applied, all of them were found to progressively increase with the enhancement of light intensity (Figure 2A). This is understandable because in this study, higher light intensity tended to produce higher standard deviation (SD) on the biomass accumulation of choy sum seedling (Table 3), leading to higher RSS and AIC accordingly. Compared to the 2D control (i.e., linear model), the AIC, RSS, and *p*-value (Prob > *F*) of the other three models were significantly lower, meanwhile their significantly higher *R*^2^ further proved their superiority (Figure 2B). Furthermore, *F*-test results of all the three models were shown to be significant as compared to that of the linear model, indicating that nonlinear models such as logistic, Gompertz and exponential ones had suitable structure for describing the 2D time-based growth of choy sum seedling and would not generate an overfitting issue (Table 2). Among them, the 2D logistic model was selected because it explicitly expressed the maximal DW achievable, superior to the other two (Figures 2A,B). From the model, the time to reach half of *DW*_*max*_ under all lighting intensities was more than 10 days, indicating that accelerated growth of this species commonly occurred at the later stage of seedling growth (Figure 7C and Table 4).

The selection criterion for the optimal 3D light-time-biomass response model was similar to that of 2D one. However, the issue of light saturation point needed to be accounted for. The light saturation point was around 400 μmol/(m^2^⋅s) and choy sum’s growth would be inhibited beyond the point, leading to a negative effect of higher light intensity on the growth. Based on the experimental data obtained in this study, two regions were identified. In Region 1, both light intensity and duration had a positive impact, whereas in Region 2, light intensity had a negative impact while duration had a positive one (Table 3 and Figure 7B). The growth model for each of the two regions should therefore be developed.

For Region 1, the LogisticCum and GaussCum models yielded significantly lower AIC, RSS, MAE, and RMSE values compared with those of Plane1 (3D control) and Exponential2D, while these two models as well as the Exponential2D one all produced significantly higher *R*^2^ and *F*-value, and significantly lower MSE compared with those of Plane1 (Figures 2C–F and Table 2). Although *F*-test results of all the 3D models were significantly higher than that of the Plane1 model, indicating that the LogisticCum and GaussCum models were better in describing the choy sum seedling growth in Region 1. Nevertheless, the LogisticCum model was chosen as the preferred model because it also explicitly indicated the maximal DW achievable in the cultivation system while the GaussCum one did not.

For Region 2, however, the light intensity applied was above choy sum’s light saturation point, and overheating was also produced due to higher temperature (29–30°C) generated under the higher light intensity environment [400–500 μmol/(m^2^⋅s)], both of which inhibited the DW accumulation of this species. Thus, the optimal plane model (Plane2), as compared to all the other models tested, yielded the highest *R*^2^ (0.850), as well as the lowest AIC, MAE, MSE, and RMSE (42.8 mg/seedling), (Figures 2C–F and Tables 2, 6). Within this plane model, the coefficient *k*_5_ (+30.47) related to time was positive while *k*_6_ (–0.46) pertaining to light intensity was negative, revealing that longer cultivation duration could still improve the growth but stronger light intensity would suppress the accumulation of choy sum DW once the light intensity was within 400–500 μmol/(m^2^⋅s) and the cultivation time was from 14 to 16 days. Taking a further examination, it was found that the negative effect of light intensity above 400 μmol/(m^2^⋅s) exceeded the positive influence of cultivation time until the 16th day on the choy sum growth, resulting in an overall decrease of choy sum DW within this range (i.e., Region 2). R2 is marked in Figure 7B.

**TABLE 6:** The robustness tests of 3D light-time-biomass response model of choy sum seedling.

Model	Region	Variable	Parameters			ANOVA		Statistics		
			Coefficient	*t*-value	Prob > | *t*|	*F* value	Prob > *F*	^*a*^*Adj. R^2^*	^*b*^*RSS*	^*c*^*RMSE*
3D light-time-biomass response model	1	Light-intensity-based model (This model)	^*d*^*DW*_0_	106.956	0	4,311*	0*	0.993	32.638*	16.3*
			^*e*^*DW*_*max*_	23.890	0					
			*t*_*m/4*_	91.392	0					
			*k*_3_	26.096	0					
			*I*_*m/4*_	45.129	0					
			*k*_4_	26.046	0					
	1	Light-energy-based model	*DW*_0_	4.408	2.165 × 10^–5^	4,5945	0	0.999	3.048	1.7
			*DW*_*max*_	74.910	0					
			*t*_*m/4*_	300.990	0					
			*k*_3_	85.139	0					
			*E*_*m/4*_	135.652	0					
			*k*_4_	83.642	0					
	2	Light-intensity-based model (This model)	^*f*^*DW*_*c*_	15.006	5.517 × 10^–6^	1,113*	1.9 × 10^–8^*	0.850	184.831*	42.8*
			*k*_5_	5.491	1.530 × 10^–3^					
			*k*_6_	-4.157	5.970 × 10^–3^					
	2	Light-energy-based model	*DW*_*c*_	15.974	3.822 × 10^–6^	1,278	1.3 × 10^–8^	0.899	161.048	12.7
			*k*_5_	5.893	1.060 × 10^–3^					
			*k*_6_	-4.306	5.060 × 10^–3^					

*^*a*^*Adj*., adjusted.*

*^*b*^*RSS*, residual sum of squares.*

*^*c*^*RMSE*, root mean square error.*

*^*d*^*DW*_0_, dry weight of choy sum seed portion that will be developed into the seedling.*

*^*e*^*DW*_*max*_, the maximal dry weight achieved.*

^*f*^*DW*_*c*_, the coordinated dry weight in Region 2. *RMSE* unit for 3D light-time-biomass response model is “mg/seedling.” *Indicated that the data were also shown in Table 2 and Figure 2F for comparison.

Logistic model has been widely applied in predicting the growth, dry matter accumulation and yield of many crops such as maize, barley, and *Solanaceous* vegetable like tomato (Prasad and Kailasam, 1992; Overman and Scholtz, 2002; Karadavut et al., 2010; Sari et al., 2019). However, its application to *Brassica* vegetables has not been reported. In this study, the 2D time-based growth models of choy sum seedling under various light intensities were all fitted well to the logistic model (Figure 7C), which agreed with those reported in the literatures on modeling the growth of other plants under constant environmental conditions such as light and soil, as well as limited nutrient supply (Milani et al., 2016; Chen, 2019; Rafiee and Mahbod, 2020).

However, the 2D logistic models for predicting growth as reported in the literature only describe the relationship between biomass and cultivation duration (Karadavut et al., 2010; Sari et al., 2019). To the best of our knowledge, this is the first time that a 3D light-time-biomass response model has been developed for the light-time relationship of vegetable growth. The 3D light-time-biomass response models clearly show that light intensity and time exerted a combined and positive influence on the biomass accumulation of choy sum seedling when below the specific light intensity of 400 μmol/(m^2^⋅s) or below 14 days of cultivation duration, but the effect of light intensity became negative once the light intensity was above the specific value (Table 5). From the 3D LogisticCum model, combining longer cultivating duration and higher light intensity but below their threshold values could achieve higher dry mass of choy sum seedling, while other combinations of longer cultivating duration with lower light intensity or shorter duration with higher light intensity would not be favorable (Table 5). It is also worth to note that this 3D LogisticCum model can be reduced to the 2D time-based growth (logistic) model under a constant light intensity condition.

Furthermore, these 3D models prove that it is feasible to develop high-quality models for predicting the growth of choy sum seedlings cultivated in indoor farm settings based on light intensity and time, once the other growth conditions such as soil types, water use efficiency (WUE) and nutrient application are fixed. Subsequently, growth conditions can be systematically optimized based on the models.

### Validation of 3D Light-Time-Biomass Response Model

**FIGURE 8:**
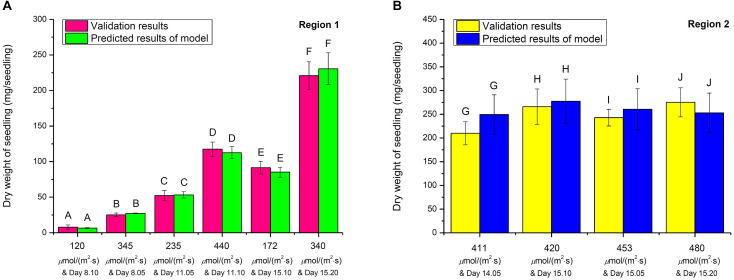
The validation studies on 3D light-time-biomass response model in **(A)** Region 1 and **(B)** Region 2. The same upper cases (A, B, C, D, E, F, G, H, I, and J) between respective validation result and predicted result of model indicate that there were no significant differences of dry weights among all the selected combinations of light intensities and duration in Region 1 and Region 2, respectively (*n* = 6, Student’s *t* test, *p* < 0.05).

The validation experiments were conducted under randomly selected growth conditions to validate the 3D model in both Regions 1 and 2. In Region 1, choy sum seedlings were grown under the randomly selected 120, 345, 235, 440, 172, and 340 μmol/(m^2^⋅s) and harvested on Day 8.10, 8.05, 11.05, 11.10, 15.10, and 15.20, respectively, while in Region 2, the seedlings were grown under the randomly selected 411, 420, 453, and 480 μmol/(m^2^⋅s) and harvested on Day 14.05, 15.10 15.05, and 15.20, respectively. The experimental seedling DW values thus collected formed a validation data set which was subsequently compared with the model predicted DW values under the corresponding growth conditions (Figures 8A,B). The results showed that there was no significant difference between all the experimental values and their correspondingly model predicted values, proving that the 3D model can reliably describe choy sum DW accumulation under a combined growth condition of light intensity and duration.

### Robustness Analysis of 3D Light-Time-Biomass Response Model

Using model variation test (Neumayer and Plümper, 2017), the robustness of 3D light-time-biomass response model of choy sum seedling was analyzed by replacing the regressor/independent variable such as “light intensity (*I*, photon numbers)” with “light energy (*E*_*I*_, Joule)” in the model and checking whether all the parameters involved in the new light-energy-based model were still strong enough to explain the dependent variable (*DW*) as the light-intensity-based 3D model did, through *t*-test. This is because light energy [J/(m^2^⋅s)] is positively correlated with light intensity [μmol/(m^2^⋅s)] generally, which makes it suitable for the substitution (Table 1). The results showed that all the parameters involved, including *DW*_0_, *DW*_*max*_, *t*_*m/4*_, *k*_3_, *E*_*m/4*_, and *k*_4_ in Region 1 and *DW*_*c*_, *k*_5_, and *k*_6_ in Region 2, in the light-energy-based model, were also significant enough to explain *DW* by showing that their respective Prob > | *t*| were remarkably lower than 0.05, the same as those of 3D light-time-biomass response model also shown in Table 6. In addition, both model significances (Prob > *F*) were also found through ANOVA (*p* < 0.05). Moreover, both ideal *Adj. R^2^* and lower enough *RSS* and *RMSE* were presented, indicating that such 3D model was still robust enough to predict choy sum seedling *DW* under different light energy, in spite of the substitution of major independent variable. In fact, 3D light-time-biomass response model is more practicable as researchers/farmers are more familiar with light intensity, rather than light energy, in the real cultivation practice, although some of indices in the light-energy-based model are better than those in the light-intensity-based one (Table 6).

## Conclusion

The growth of choy sum seedling is strongly dependent on light intensity as we hypothesized. Using LED lighting technology, the optimal range of illumination conditions to cultivate choy sum seedling was found to be around 400 μmol/(m^2^⋅s), under which the largest leaf area, the best stem architecture with the biggest hypocotyl diameter and the shortest hypocotyl length, the most well-developed root system with the longest root length and the highest root biomass, and a satisfactory photosynthetic efficiency were achieved. The 3D light-time-biomass response model developed can be utilized for reliably predicting the productivity of choy sum seedling in indoor plant factory under inhomogeneous distribution of illumination level of indoor farm racks, by showing excellent model robustness. Such model is convenient and practicable to be applied in indoor farming, because the researchers/farmers would just need to estimate the growth duration, as well as the light intensity of the site where choy sum seedling grown. Once all the other environmental factors such as temperature, soil pH and nutrients, along with CO_2_ and water are settled and stable, then they could accurately calculate the biomass of choy sum seedling, which allows them to efficiently evaluate choy sum seedling production in indoor plant factory. Future research should be focused on the impacts of photoperiod and red blue light ratio on the growth of choy sum seedlings in order to further optimize energy conversion efficiency in plant factories.

## Data Availability Statement

The original contributions presented in the study are included in the article/supplementary material, further inquiries can be directed to the corresponding author.

## Author Contributions

JH: conceptualization, methodology, investigation, data collection and processing, writing–original draft, and writing–review and editing. CD’S: methodology, investigation, and writing–review and editing. WZ: conceptualization, methodology, writing–review and editing, supervision, and project administration. All authors contributed to the article and approved the submitted version.

## Conflict of Interest

The authors declare that the research was conducted in the absence of any commercial or financial relationships that could be construed as a potential conflict of interest.

## Nomenclature

**Table:**

*μ*	Relative growth rate (/day)
*A*	Avogadro constant (6.022 × 10^23^ No./mol)
*AIC*	Akaike Information Criterion
*c*	Light ray velocity (2.998 × 10^8^ m/s)
*DW*	Dry weight (mg)
*E*	Energy (J)
*FW*	Fresh weight (mg)
*h*	Planck constant (6.626 × 10^–34^ J⋅s)
*I*	Light intensity [*μ*mol/(m^2^⋅s)]
*k*	Coefficient of model
*k*_1_	Light-intensity-related coefficient of *P*_*n*_ [(m^2^⋅s)/g]
*k*_2_	Time-related coefficient of 2D time-based growth model (/d)
*k*_3_	Time-related coefficient of 3D light-time-biomass response model (d) in Region 1
*k*_4_	Light-intensity-related coefficient of 3D light-time-biomass response model [*μ*mol/(m^2^⋅s)] in Region 1
*k*_5_	Time-related coefficient of 3D light-time-biomass response model (mg/d) in Region 2
*k*_6_	Light-intensity-related coefficient of 3D light-time-biomass response model (mg⋅m^2^⋅s/*μ*mol) in Region 2
*LDW*	Leaf dry weight
*LED*	Light Emitting Diode
*M*	Dry mass of seedlings per area (g/m^2^)
*MCP*	Moisture content percentage (%)
*N*	Number of experimental data points
*NAR*	Net assimilation rate [g/(m^2^⋅d)]
*p*	Total number of parameters included in the model
*PDY*	Productivity [g/(m^2^⋅d)]
*PE*	Photosynthetic efficiency (%)
*P*_*max*_	Maximal *P*_*n*_ [g/(m^2^⋅s)]
*P*_*n*_	Net photosynthetic rate [g/(m^2^⋅s)]
*R*^2^	Coefficient of determination
*RSS*	Residual sum of squares
*TLA*	Total leaf area (m^2^)
*TSS*	Total sum of squares
**Subscripts**	
*B*	Biomass
0	Day 0 counting from sowing
*I*	Light intensity
*m/2*	Light intensity/time to reach half of the maximum
*m/4*	Time/light intensity/light energy to achieve 25% of *DW*_*max*_
*max*	The maximal value
*n*	Day *n* counting from sowing
*t-I*	Time and light intensity related
*λ*	Photon wavelength
